# Peering in: youth perspectives on Health Promoting Schools and youth engagement in Nova Scotia, Canada

**DOI:** 10.1093/heapro/daac081

**Published:** 2022-07-21

**Authors:** Julia C Kontak, Hilary A T Caldwell, Margaret Kay-Arora, Camille L Hancock Friesen, Sara F L Kirk

**Affiliations:** Faculty of Health, Dalhousie University, Dalhousie University, 5968 College Street, PO Box 15000, Halifax, NS B3H 4R2, Canada; Healthy Populations Institute, Dalhousie University, 1318 Robie Street, Halifax, NS B3H 3E2, Canada; Healthy Populations Institute, Dalhousie University, 1318 Robie Street, Halifax, NS B3H 3E2, Canada; Healthy Populations Institute, Dalhousie University, 1318 Robie Street, Halifax, NS B3H 3E2, Canada; Healthy Populations Institute, Dalhousie University, 1318 Robie Street, Halifax, NS B3H 3E2, Canada; Division of Pediatric Cardiothoracic Surgery, Children’s Health and Medical Centre, University of Nebraska Medical Center, 8200 Dodge Street, Omaha, NE 68114, USA; Healthy Populations Institute, Dalhousie University, 1318 Robie Street, Halifax, NS B3H 3E2, Canada; School of Health and Human Performance, Dalhousie University, PO Box 15000, Halifax, NS B3H 4R2, Canada

**Keywords:** Comprehensive School Health, school health, health promotion, youth participation, health education

## Abstract

Health Promoting Schools (HPS) is a whole-school approach that shapes the conditions necessary to support student health and well-being. Youth engagement is recognized as key to HPS implementation, yet research related to the involvement of youth voice in school health promotion initiatives is limited. The purpose of this study was to understand youth perspectives on HPS and school youth engagement. Ten youth (grades 9–10, ages 14–16) were trained as peer researchers using a Youth Participatory Action Research approach. The peer researchers interviewed 23 of their peers (grades 7–10, ages 12–16) on perspectives related to HPS and school youth engagement. All interviews were audio-recorded, transcribed and data were analysed using inductive ‘codebook’ thematic analysis. Themes related to a healthy school community were mapped onto the pillars of HPS: (i) Social and Physical Environment, (ii) Teaching and Learning, (iii) Partnerships and Services and (iv) School Policies. Participants placed more importance on the social and physical environment of the school including respect, inclusivity, supportive relationships and the design of spaces. Key factors for youth engagement were: (i) safe and supportive spaces, (ii) passion and interest, (iii) using their voice, (iv) power dynamics, (v) accessibility and (vi) awareness. With recognition that youth engagement is a crucial part of HPS, this work provides relevant and applicable information on areas of the healthy school community that are important to youth, and if/how they are meaningfully engaged in school decision-making.

## Graphical Abstract

**Figure:**
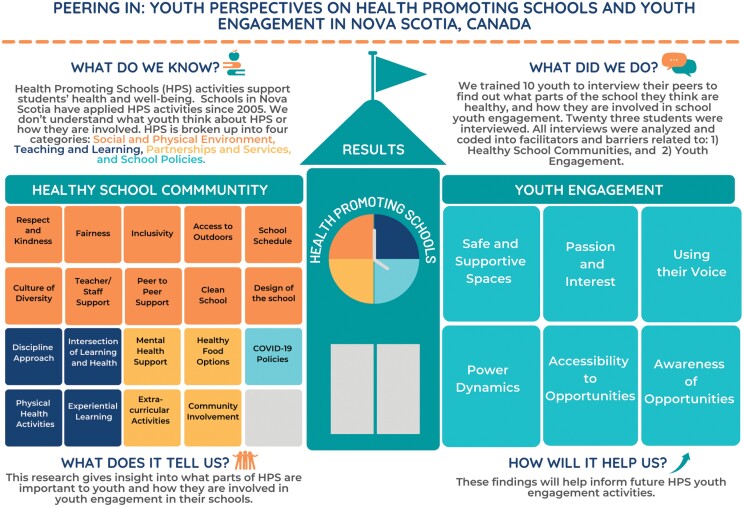

## BACKGROUND

Ever since 1986, when the Ottawa Charter for Health Promotion described that settings where people live, work and learn significantly impact their health (World Health Organization [WHO], 1986), school environments have been viewed as a key setting for students to develop and adopt health behaviours (Tjomsland *et al.*, 2009; Penney *et al.*, 2017; Gugglberger, 2021).

Health Promoting Schools (HPS), also referred to as Comprehensive School Health, is a globally recognized whole-school approach that puts concerted efforts into shaping the conditions necessary to support children and youth’s health and well-being rather than focussing on individual health behaviours (WHO and UNESCO, 2021). When evaluated, HPS efforts have demonstrated promising, but mixed effects, including increases in physical activity (Fung *et al.*, 2012; Langford *et al.*, 2015), improvements in healthy eating (Fung *et al.*, 2012; Langford *et al.*, 2015), enhancement of personal development skills (Stewart-Brown, 2006), a decrease in bullying behaviours (Langford *et al.*, 2015) and positively influencing educational outcomes (Lee *et al.*, 2006; Suhrcke and De Paz Nieves, 2011). HPS approaches also have economic value, primarily through future health care cost savings (Tran *et al.*, 2014). In Nova Scotia, Canada the HPS approach was adopted provincially in 2005 and includes four inter-related pillars: (i) Social and Physical Environment; (ii) Teaching and Learning; (iii) Healthy School Policy and (iv) Partnerships and Services (Province of Nova Scotia, 2015; Pan-Canadian Joint Consortium for School Health, 2018). However, the implementation of the approach over the past 16 years has been inconsistent across school communities, including lack of rigorous evaluation and youth engagement opportunities (McIsaac (Langille) *et al.*, 2016; McIsaac *et al.*, 2017). This is concerning as the need to collaborate with community partners to develop and enhance healthy school initiatives is widely recognized as valuable (Griebler *et al.*, 2017), yet research related to the benefits of youth engagement in school health promotion is still limited (Beck and Reilly, 2017; Griebler *et al.*, 2017; Sprague Martinez *et al.*, 2020).

As top-down whole-school approaches show mixed effects (McIsaac (Langille) *et al.*, 2016; McIsaac *et al.*, 2017), it is advantageous to engage youth in school health promotion activities to develop more relevant and applicable strategies to meet their target needs. The inclusion of student voice in HPS initiatives aligns with the principles of the United Nations Conventions on the Rights of the Child (United Nations, 1989). Specifically, Article 12 and 13 of the Conventions refers to the capability and participatory right of children to have their own views, to express their views and for their views to be duly considered (United Nations, 1989; Lundy, 2007). Youth participation in schools can be considered a partnership or collaboration between students and diverse adult stakeholders (Griebler *et al.*, 2017). The overarching aim of youth engagement is to meaningfully engage youth in decision-making that leads to changes in school programmes and policies (Sprague Martinez *et al.*, 2020). Various benefits of youth engagement have been outlined for youth, adults and organizations. For example, youth can develop life skills, and improve personal development, motivation and civic engagement (Centre for Excellence in Youth Engagement, 2003; Mitra, 2004, Zeldin, 2004; Mager and Nowak, 2012; Griebler *et al.*, 2017), while adult champions have reported improvement in their understanding of youth and quality of their relationships with young people (London *et al.*, 2003; Mitra, 2004; Griebler *et al.*, 2017). At a systems level, organizations and institutions are shown to be more responsive to youth needs through more relevant policies, programmes and initiatives (Zeldin, 2004; Griebler *et al.*, 2017). Different youth engagement spectra have been developed over the years to understand how and to what degree youth are being engaged in decision-making. The spectrum of youth engagement was first outlined by Hart’s Ladder of Participation (Hart, 2008). The steps on the ladder represent increasing degrees of youth engagement beginning with nonparticipation (i.e. manipulation, decoration and tokenism) and extending through five degrees of involvement (i.e. shared decision-making).

Despite a growing practice and acknowledgement about the importance of youth engagement within the school setting, there is limited research understanding how youth are engaged in decisions related to healthy school initiatives (Griebler *et al.*, 2017; Larsson *et al.*, 2018; Sprague Martinez *et al.*, 2020), specifically whole-school health approaches like HPS (Simovska and Carlsson, 2012). Limited research on involvement of youth in school health promotion is concerning, as meaningful youth engagement is recognized as key to implementation of HPS approaches (Storey *et al.*, 2016; Canadian Healthy Schools Alliance, 2021; WHO and UNESCO, 2021).

With youth engagement being the topic of inquiry, a Youth Participatory Action Research (YPAR) approach was used for this study. YPAR is an approach that conducts research *with* youth rather than *on* them, by engaging young people in different stages of the research process to address social problems through youth–adult partnerships (Anyon *et al.*, 2018). Having youth involved in the collection, analysis, interpretation and/or dissemination of research can provide value to and applicability of the research findings (Jacquez *et al.*, 2013; Ozer, 2017). The YPAR approach also provides positive outcomes in and of itself to youth, including agency and leadership, academic or career achievement, social, interpersonal and cognitive development, and strong relationships with adults (Shamrova and Cummings, 2017; Anyon *et al.*, 2018). YPAR is an increasingly common approach to enhance youth development and actively engage young adults within decision-making processes (Soleimanpour *et al.*, 2008; Anyon *et al.*, 2018).

To our knowledge, there are limited studies that examine whole-school health approaches from youth perspectives, although there are studies that provide youth views on specific school health issues (Larsson *et al.*, 2018), such as food environments (Spencer *et al.*, 2019), bullying (Ybarra *et al.*, 2019), race (Bañales *et al.*, 2021) and sexual health (Soleimanpour *et al.*, 2008). While it is helpful to study various health issues independently, it limits the ability to understand what areas of a healthy school community are most important to students. Given the limited research and proposed benefits of youth engagement in HPS activities, the purpose of this research was to understand youth perspectives on HPS, as well as how they are engaged in school decision-making.

## METHODOLOGY

### Youth Participatory Action Research

This study took a YPAR approach by involving youth who represented various populations and locations across Nova Scotia, Canada to participate in the project as peer researchers. Youth (grades 9–10, ages 14–16) who participated as peer researchers took part in 2 days of in-person training in the summer of 2021. Members of the research team with expertise in youth engagement, research methods and participatory research facilitated the training and delivered the workshop on the following topics: HPS pillars, healthy school environments, participatory research, how to conduct semistructured interviews, principles related to ethical conduct of research (e.g. mitigating conflict of interest and avoiding coercion), and how to use the digital recorders. Youth completed practice interviews with the other peer researchers to develop their comfort with the interview guide and digital recorders. Peer researchers were given the digital recorders at the end of training. Youth then conducted semistructured interviews with their peers to understand their perspectives on HPS, as well as their engagement in school decision-making processes. Youth researchers were responsible for recruiting peers, collecting consent forms and administering the interviews. This paper focusses on the process and findings of the interviews the peer researchers conducted.

### Participants

This study used a convenience sampling method as peer researchers and interviewees were selected based on the availability and willingness to take part in the study (Creswell and Poth, 2016). Members of the research team recruited participants for this training. Parents and youth serving organizations were contacted based on previously established relationships with the research team members. Detailed explanations of the training were provided to parents and youth serving organizations including a promotional poster on the purpose of the research, and role of the youth as peer researchers. Once youth expressed interest, communication occurred directly with them and in some cases, youth assisted with recruiting additional participants. Ten peer researchers were recruited for training with eight identifying as a woman and two identifying as a man. Two identified as French Acadian, six as New Immigrants and two as white Canadians. Peer researchers were asked to identify 2–3 of their peers to interview and were encouraged to select a diverse group of young people that ranged in age (grades 7–10, ages 12–16) and experiences. Besides being a student in grades 7–10 and attending a public school in Nova Scotia, Canada, there were no other inclusion criteria.

### Materials

The semistructured interview guide (referred to as ‘guide’) was codeveloped by the research team, with input from school health promotion staff, before being reviewed and edited by the peer researchers. A draft guide developed by the research team and adult supporters was presented to the peer researchers during their training and they were asked about the content, approach and suitability of the guide for application with this age group. Peer researchers provided feedback that was incorporated into the final version of the guide. The guide (Appendix A) included a short summary of what the research project was about, how privacy and confidentiality would be maintained, a paragraph and a visual depiction of the HPS model, and open-ended questions with supplementary prompts. The guide was separated into three sections: (i) Healthy School Communities: What does it mean, (ii) Health School Communities: What’s important to you and (iii) Youth Voice and Leadership. Examples questions included: ‘*What is your school doing well to be a healthy school community?*’ and ‘*How could students be more involved in decision making at your school?*’.

### Data collection

The peer researchers were supplied with a consent form, an information sheet and the semistructured interview guide. The information sheet provided helpful reminders for the peer researchers as well as information for them to share with participants including: the purpose, risks/benefits of the interview, privacy concerns, information on the length of the interview and information on who to contact in case of a question (Appendix A). Interviews were conducted virtually or in-person depending on public health guidelines in place at the time due to the COVID-19 pandemic, as well as participant preference. Peer researchers provided the consent form to the interviewee and ask them to review and sign the form prior to participating in the interview process. Peer researchers reviewed the information sheet, and the visual depiction of the HPS approach before beginning the interview. All interviews were audio-recorded and took between 30 and 60 min to complete. Instructions and recording devices were provided to peer researchers. Peer researchers were instructed to only record the interviews on the recording devices provided to them at the training session.

### Ethical considerations

Ethics was approved prior to peer researcher training and data collection from the Social Sciences and Humanities Research Ethics Board at Dalhousie University, Nova Scotia, Canada [2021-5701]. As the peer researchers and interviews were both under the age of majority, parental/guardian consent was obtained for both groups. For the peer researchers, parental/guardian consent was obtained at the time of training. For interviewees, parental/guardian consent was obtained prior to the interview by the peer researcher. Peer researchers received a stipend or their participation in the role to recognize their contribution at $13.50/h (CAD), which was slightly above the provincial minimum wage at the time of the interviews. Interviewees were provided a $15 (CAD) gift card for their time.

### Data analysis

Audio-recordings from peer researchers were sent back to a member of the research team. Interviews were transcribed verbatim, and all data and quotes were dedentified, with participant names replaced by pseudonyms. All interviews were imported and analysed in Nvivo 12 Qualitative Analysis Software (Qualitative Data Analysis Software | NVivo, n.d.).

A pragmatic approach was taken to the data analysis, such that the analysis was guided by what best met the needs of the topic of inquiry and the practical implications of research (Creswell and Poth, 2016). The pragmatic approach instilled post-positivist principles (Creswell and Poth, 2016) to provide a more logical structure to the complex model of HPS. With this paradigm in mind, an inductive ‘code-book’ thematic analysis (Braun and Clarke, 2021a, b) was used to analyse the interviews. An inductive ‘codebook’ thematic analysis provides structure for documentation through the development of initial themes, but also provides room for refinement, adaption and development of new themes throughout the analytical process (Braun and Clarke, 2021a, b). As the data were collected by peer researchers rather than by the researcher who analysed the data (J.C.K.) this method is appropriate as it provides the ability to stay closely linked to the direct dataset, but the opportunity for refinement through the analysis process.

To begin, the researcher (J.C.K.) coded the first five transcripts and used these transcripts to develop a codebook that consisted of overarching themes, code names, brief definitions, when to use the code (or not), and examples of each (MacQueen *et al.*, 1998). This codebook was used as a guide, and aided in confirmability (Korstjens and Moser, 2018) across the analysis process.

The initial themes and subthemes were presented to the research team (H.A.T.C., M.K.-A., C.L.H.F. and S.F.L.K.). Refinement of themes and subthemes were made based on the discussions. Due to congruency of some themes to the HPS model, it was decided to map these themes onto the four inter-related pillars of the model (Province of Nova Scotia, 2015). Subthemes were further separated into enablers and barriers to support interpretation of findings and ensure they are accessible to different stakeholder audiences.

Multiple techniques to enhance trustworthiness were instilled including: (i) In-depth training and resources provided to peer researchers, integration of feedback on the data collection tool, and refinement of preliminary themes with research team members for credibility purposes; (ii) A detailed track record of peer researcher training and data collection methods, development of an analytic plan and iterative codebook for dependability; (iii) Regular debriefing and integration of edits from the research team for confirmability and (iv) Effort to recruit a diverse group of peer researchers and interviewees and a description of the participants, materials and analysis to enhance transferability of the research findings. Limitations of the research study can be reviewed in *Discussion*.

## RESULTS

Twenty-three youth were interviewed by the 10 trained peer-researchers with each peer researcher conducting 2–3 interviews. Six interviewees reported they were in grade 7, three in grade 8, seven in grade 9 and seven in grade 10. Other demographic information of participants was not collected. Results are presented across two main areas of focus: (i) factors related to a healthy school community and (ii) factors related to youth engagement within the school.

### Healthy school communities

Identified themes, subthemes and direct quotations related to factors that are facilitators or barriers to a healthy school community are outlined in Table 1. Subthemes were categorized into the HPS framework (Province of Nova Scotia, 2015), including: (i) The Social Environment and Physical Environment, (ii) Teaching and Learning, (iii) Partnerships and Services and (iv) School Policies.

**Table 1:** Factors related to a healthy school community

Themes	Facilitators	Barriers	Quotes^a^
Social and Physical Environment			
Respect and kindness	Happy, friendly, safe and enjoyable atmosphere.	Physical (fights) and verbal bullying and violence (teasing, negative comments).	‘*…all students are respected, they respect each other, they respect the teachers and the principals and stuff… and no bullying, so no violence in the school.*’
Fairness	Acts of fairness within the school.	Favouritism by the teachers and staff.	‘*…not all the teachers, but some of the teachers, you can notice they don’t treat all of the students the same, or fairly…*’
Inclusivity	Nonjudgemental setting that is supportive and accepting.	Judgement and not feeling a sense of belonging.	‘*…less judgemental, I feel like that is a big thing because like everyone is going to have a little shell and then if there are judgemental people they’re going to stay in the shell. But when there are people, they don’t feel like judge them they open up.*’
Culture of diversity	Acknowledgement of the importance of diversity and that racism should not be condoned.	Racism is prevalent and related to acts of violence and bullying.	‘*…racism, racism is a very serious thing. It is very important to know that every black life matters. We need more black people to talk about their experience with racism and make it aware that it is not acceptable.*’
Teacher/staff support	Trusting and supportive relationships that actively listen and are observant.	Neglecting ideas or not prioritizing issues that are important to youth.	‘*I think the adult’s main role, I think, it’s important for them to be supporting their students, to be listening, to be helpful, and for them to again, encourage everyone to discuss, to share their thoughts…*’
Peer-to-peer support	Positive and supportive relationships that listen, observe and check-in on one another.	None Identified.	‘*…when I first arrived, when it was my first day, I felt really welcomed, and like everyone was making an effort to make me feel welcomed and for me to have friends.*’
Access to outdoors	Ability to learn and play outdoors, as well as have access to fresh air.	Lack of windows.	‘*Especially in our school where there is not a lot of windows. That is not healthy. They should hit some bricks and make holes in the walls. A big wrecking ball right through the wall. More fresh air.*’
School schedule	Longer days, with more breaks for lunch, movement and free time.	Structure of the school day including length, break schedules and the content taught.	‘*I think especially for our school since we end at 1:55 pm, there is a lot more time in the day so we could have another half hour for lunch or more time for outside and in between classes or bigger breaks. We would get less stressed.*’
Clean school	None identified.	Garbage, lack of air flow (i.e. air conditioning) and food waste.	‘*People pick up their garbage, throw it in the trash can. Empty out their desk if they have food in there and keep everything clean. Check it every week.*’
Design of the school	Collaborative, large, bright and well-designed spaces that are conducive to learning including more modern classrooms, comfortable and shared seating arrangements, lots of windows and appealing décor.	Out-dated architecture (e.g. no windows), small classrooms compared with the number of students and budget for updates to school environment.	‘*I really liked that there was lots of comfortable seating in my classroom, there were couches, and all of our tables and chairs were new. There were highchairs, spinny chairs I think, also the chairs with bouncy balls as the seat would be really nice for people, especially if they’re very fidgety and if it helps them learn.*’
Teaching and Learning			
Discipline approach	Necessary when a rule is broken (e.g. mask mandates) or when a student needs to be accountable for their actions.	Being too strict to certain students or when there are too many rules.	‘*…like people definitely have trouble with administration, that’s one thing, they can, for some, the areas they might be a bit too strict, and suspensions are sometimes kind of thrown around…*’
Intersectionality of learning and health	Acknowledgment of embedding health activities into traditional education.	Physical activity generally viewed as separate from regular learning yet still seen as important.	‘*Oh and for physically being active, maybe every morning, a little excitement doesn’t kill people. Put on a funny show, or exercise. So you tell all the kids to stand up and do what the exercise is doing, you know. Make it funny, so it makes the person wake up every morning after being so tired.*’
Experiential learning	Learning is enjoyable, fun and collaborative.	None identified.	‘*…if our schools have more celebrations and activities, our entire school gets involved, interacts, I think it will be more fun and people have more excitement to go to school.*’
Physical health activities	Physical activity throughout the school day and a range of activity types.	Limited time for gym class.	‘*And for gym class, we won’t do that much running and soccer ball, we’ll do more fun games, like physical activities, not like real sports and stuff…*’
Partnerships and Services			
Mental health support	Receiving support and resources from peers, staff and teachers.	Additional staff members needed (e.g. guidance counsellors, educational programme assistant) to assist with youth needs.	‘*For example, on Bell Let’s Talk Day, our homeroom teacher initiated a discussion about mental health, and she gave us many resources if we needed them, such as, I think it was called… Kids Help Phone, which you can text anytime. And she really made us feel like we could talk to her if we needed to, or any other teacher.*’
Extra-curricular activities	Opportunities to foster connection across different peer groups.	Lack of leisure activities and desire for more extra-curriculars.	‘*A student body where it feels like everyone has a part they can play, so like, there’s clubs that you can be involved in, there’s a sport you could find that you’re interested in, or… there’s something like student council where you can get involved.*’
Healthy food options	Healthy food available at school through breakfast and lunch programmes.	Expensive food options.	‘*…I feel like you should maybe introduce school lunches, maybe because like some parents wake up early in the morning to make their children lunches and they have to go to work and it’s all like very frustrating… So I feel like you should bring meals like lunches and they should be healthy because many people go out for lunch, go to McDonalds. I mean that is fine but at some point it’s not too healthy but if you bring a healthy meal it would help.*’
Community involvement	Guest speakers presenting on specific topics of interest.	Limited knowledge on how community or parents/family are involved.	‘*Also, maybe guest speakers talking about important topics that could really teach you and really make you realize that you believe in the same cause, or you believe in the same issue that needs to be changed.*’
School Policies			
COVID-19	Added outdoor breaks and more opportunities for fresh air.	Socialization with their peers, specifically those in other classes.	‘*It has gotten better but at the start of the year, we had zones, we couldn’t go out for lunch and had to eat in our classroom.*’

Any grammatical errors in direct quotes were edited by research team for clarity.

It was evident from the youth interviews that a social environment is a vital part of a healthy school community. Youth described a healthy school community as a setting with a social environment that is safe, enjoyable, happy and where people are kind and respectful to one another. Within this social environment, a desire for a safe, fair, diverse and accepting community was broadly indicated; however, this was not always reported as a reality that participants experienced in their current school context. Some youth reported experiencing acts of racism, observations of favouritism and the presence of bullying behaviours (verbal and physical). It was acknowledged by participants that these acts of discrimination and violence were not condoned, and strategies to mitigate these behaviours, such as building awareness and having the opportunity to talk through concerns were shared. Further, youth particularly voiced the importance of inclusivity, indicating that a setting that is nonjudgemental, and accepting is a central component to creating a healthy school community, while a lack of sense of belonging or ‘place’ hinders an inclusive setting. Supportive relationships between teachers and students, as well as peer-to-peer support were reported as essential to a healthy school environment. Youth specifically shared the significance of having trusting relationships with teachers that are understanding and observant. However, youth indicated this relationship may be impacted if they felt their ideas were not being heard or taken seriously.

Approaches to teaching and learning were also identified as key factors to influence a healthy school environment. Of particular importance was the role of experiential learning to enact a healthy school community, including the incorporation of different forms of movement (e.g. dance) and creativity (e.g. baking, knitting) into their class activities, as well as more collaborative group activities. Some youth recognized the value of embedding health activities within learning exercises such as outdoor learning and physically active learning, yet most students viewed these activities as separate from their regular learning. Regardless of this viewpoint, physical and mental health activities were still considered a vital part of student’s daily schedule.

Beyond social factors, participants described components of the physical environment that they desire in a healthy school community. Most discussed factors related to the design of the school, including brighter lights, larger windows, appealing décor (e.g. murals and student touches), as well as collaborative and comfortable workspaces. On the contrary, out-dated designs, small classrooms and lack of windows were considered to inhibit health and learning. One student shared that their school resembled a jail. Further, a clean school, including minimal garbage, and well-kept spaces, as well as access to an outdoor area, were shared as necessary components. Participants also indicated the impact of the school schedule on their health and well-being, such that changes in timing of breaks (e.g. lunch, recess) or the length of the day could provide more opportunity for health-related activities such as outdoor breaks and free time.

Lastly, youth shared factors related to the COVID-19 pandemic that impacted the health of the school environment. Generally, youth only referred to school policies and rules when referencing COVID-19 regulations, such as the requirement to wear a mask, or being limited to only interacting with students in their classroom. Youth indicated these regulations negatively impacted socializing with their peers, specifically those in other classes. In contrast, youth spoke positively to other COVID-19 procedures that led to added outdoor breaks and more opportunities for fresh air.

### Youth engagement

Participants provided perspectives related to the importance of youth voice, the process of youth engagement and factors that enabled or hindered youth to be part of school decision-making.

Youth shared that it is vital to engage students in school decisions as they are the ones who will be impacted and benefit the most. Youth indicated that their perspectives may be fundamentally different than their adult counterparts; therefore, it is critical to take into consideration different viewpoints before moving forward with a decision. As shared by a participant, ‘*I think it’s really important because the schools and the people in charge themselves, they don’t have the perspective of going to school in the community firsthand. So, I think it’s important for them to get another perspective, another opinion, and the thoughts of actual members of the school community itself.*’

Similar initiatives were described by participants when sharing their involvement in school decision-making activities. Most examples were related to informing a decision, such as voting polls, petitions and suggestion boxes. Their description of the process also alluded to their understanding of compromise, such that they acknowledged their perspectives are one part of the decision-making process and that teacher and staff perspectives also needed to be taken into consideration. As described by one youth, ‘*I liked how it was a group effort, so there was points of view from different people, and then we still had teachers to input their ideas, so then we could come together as a collective group and agree on something. So then it made the points feel a lot healthier because people were listening to our ideas, as well as we were listening to theirs*.’

Though students indicated the desire to have their voices heard more frequently rather than only informing certain decisions, a few examples provided initiatives that were student-led or a collaboration between students, teachers and staff. Examples included clubs (e.g. Change Makers Clubs), advisory boards, student councils and music/dance initiatives.

Most of the information shared by participants about youth engagement was related to what enabled or inhibited their involvement in these activities. Facilitators and barriers of youth engagement, and direct quotes from participants are outlined in Table 2 and included: (i) safe and supportive spaces, (ii) passion and interest, (iii) using their voice, (iv) power dynamics, (v) accessibility and (vi) awareness.

**Table 2:** Facilitators and barriers for youth engagement

Theme	Facilitators	Barriers	Quotes^a^
Safe and supportive spaces	Encouraging youth to share their opinions and thoughts and reinforce confidence.	Fear of ideas being ‘shut down’ or being controversial.	‘*The teachers are very helpful and supportive, they support your ideas and your thinking, and they really encourage you to again, voice your thoughts, and learn about what you think is important, and initiate change, if that’s what you believe in.*’
Passion and interest	Emotions and personal interest connected to issue and determination for change.	None identified.	‘*I liked that we were able to start a project about something that we actually believed in and we had a passion in changing, and changing for the better. So we were able to, as a class, to do something that we believed in and wasn’t initiated by a teacher that we found boring and weren’t very interested in.*’
Using their voice	Feelings of empowerment and importance that inspire continued involvement.	Not being listened to, or ideas are not acted or considered by teachers/staff.	‘*And when people ask about our feelings it makes us feel okay, they want my voice heard, they want my suggestions, I’m wanted, I’m wanted here. So it just makes the child feel better and it makes them push more to speak out when they don’t like something or if they aren’t comfortable with something.*’
Power dynamics	None identified.	Power imbalance between youth and adult counterparts, and sense of not being taking seriously.	‘*…we were a grade 6 class, so our opinions and thoughts, they wouldn’t be as respected, or, they would be respected but they wouldn’t be taken as seriously as if we were adults, or people in charge.*’
Awareness	None identified.	Lack of awareness or clarity on how to get involved.	‘*The only thing is… there’s clubs, so people can get into clubs, I think that’s the only way to get involved at school. I think there’s groups in our school that make decisions but if they don’t say then no one knows it. They should advertise it more, so more people can get involved.*’
Accessibility	More students involved, and diverse forms of engagement.	Lack of opportunities and options to be engaged at different levels (i.e. only leadership options).	‘*Definitely just making more ways for students to have an impact on the school around them. Like there might be clubs that I’m unaware of, but not necessarily only clubs just different ways a student could voice their opinion.*’

Any grammatical errors in direct quotes were edited by research team for clarity.

Youth shared that their interest in being involved in school decisions often came from their desire, motivation and passion to make change on a certain issue. Yet to do so, it was commonly indicated that it was vital to have a safe and supportive environment where they were comfortable to voice their opinions through the encouragement of students, staff and, in particular, teachers. However, it was frequently shared that an environment in which they were fearful to share their ideas due to the apprehension of being ‘shut down’ or that their opinions would be considered controversial was a barrier to engaging in the decision-making process. This fear may be connected to participants alluding to a power imbalance between students and teachers/staff, such that student’s opinions may not be respected or taken seriously compared with their adult counterparts. Strategies to mitigate these barriers were shared, including opportunities to ensure anonymity of opinions, such as suggestion boxes.

Beyond support, participants explained that feeling like their voices were heard and listened to encouraged them to continue to take part in engagement initiatives; yet there were mixed remarks on whether their opinions were acted on or considered. When listened to, youth indicated that the feeling of empowerment and importance reinforced and inspired them to be part of engagement opportunities.

Additionally, factors related to awareness of or access to decision-making opportunities were voiced as impacting engagement. Lack of communication or knowing when and where these initiatives take place in a school community were shared. Further, youth indicated that there were not enough engagement opportunities within the school community. Not all students may be interested in being part of a club or would like to take a leadership position. It was indicated that differing levels and types of opportunities for engagement may aid in recruiting a diverse group of youth to be part of these initiatives.

## DISCUSSION

This research study used a YPAR approach to train students as peer researchers and enable them to interview their peers to explore youth perspectives on HPS and school youth engagement. Results were organized into factors (enablers and barriers) that impact a healthy school community and youth engagement.

### Youth Participatory Action Research

Our YPAR approach involved youth in the data collection process by training them as peer researchers to recruit peers, collect consent forms and conduct semistructured interviews. Comparable to our study, Soleimanpour *et al.* (Soleimanpour *et al.*, 2008) used a YPAR approach by training youth as student researchers to explore health needs of their peers; resulting in successful engagement of youth and applicable findings to improve school-based health centres. Beyond peer researchers, various methods have been used to engage youth within the research process including photovoice (Wang, 2006; Spencer *et al.*, 2019), reflective writing (Sonn *et al.*, 2011) and advisory councils (Mandel and Qazilbash, 2005). Regardless of the method, YPAR has consistently shown valuable benefits to the research process (Ozer, 2017), as well as positive impacts on youth development (Anyon *et al.*, 2018). Training youth to conduct interviews is an innovative YPAR approach to engage youth across the research process as well as share responsibilities between youth and adult supporters.

### Healthy school communities

In relation to healthy school communities, students indicated the importance of all four pillars of the HPS model: (i) Social and Physical Environments, (ii) Teaching and Learning, (iii) Partnerships and Services and (iv) School Policies (Province of Nova Scotia, 2015). Greater emphasis was placed on the Social and Physical Environments pillar, with particular importance on the factors that enable or inhibit the safety and learning environment of the students including a respectful and inclusive setting, supportive relationships and the physical design and construction of the school. In general, youth were unclear how school policies (beyond COVID-19 regulations) impacted their school environment and how community partnerships are connected to the school.

A study from Prince Edward Island (P.E.I.), Canada, a province with a similar context to Nova Scotia, provides opportunity for comparison. Murnaghan *et al.* (Murnaghan *et al.*, 2013) conducted focus groups with fifty students from across the three school boards in P.E.I. to examine what students thought to be the most important health issue to study within their school. Aligning with our findings, Murnaghan *et al.* (Murnaghan *et al.*, 2013) found that faciliators included clean and well-kept school environments, healthy food options, movement and physical activity options, bullying prevention and postive support from students, teachers and staff. However, our findings suggested that youth placed greater importance on safety and mental health, while Murnaghan *et al.* (Murnaghan *et al.*, 2013) findings had minimal results related to this concept and a greater importance on healthy food and physical activity options. In a study that used reflective writing, photovoice and focus groups to understand youth perspectives on HPS, a priority on safe environments, including fairness and equality, was identified (Sonn *et al.*, 2011). However, this research was conducted in a low-income suburban school area of South Africa (Sonn *et al.*, 2011), a very different context compared with our study. The emphasis on safety and mental health found in our study’s findings compared with past research may be due to the overall focus and recognition of the importance of these two components in schools over the past decade—leading to adaptive perspectives on what youth view as important factors of a healthy school community. A recent scan of HPS policies in Nova Scotia reiterates this possibility highlighting that ‘safe school environment’ and ‘equity, inclusivity and accessiblity’ policies were the most commonly identified, while ‘physical activity’ and ‘nutrition’ were among the lowest (Graham-DeMello *et al.*, 2021). However, this scan also indicated that mental health policies were minimal as well. It may also have been related to the context of the COVID-19 pandemic, which was ongoing at the time the data were collected, since this included a focus on safety with respect to school-level public health protocols. Although students in our study did not mention particular policies related to safe environments and mental health, there was emphasis on the importance of developing a culture of safety, inclusivity and respect which may also be partially influenced by the current political focus on student safety in Nova Scotia (Whitley and Hollweck, 2020).

The lack of participant awareness of school policies beyond COVID-19 regulations suggests that students may not understand or be readily involved in the the full decision-making process related to school health and well-being. Similar to our findings, case study research conducted in the Macedonian Network of Health Promoting Schools in Europe outlined a collaboration between students, teachers/staff and school health promotion consultants to shorten the class schedule to free up more time for sports, socializing and relaxation (Bruun Jensen and Simovska, 2005). This study highlighted the need for students to understand the policy process given the approval and buy-in that is necessary from leadership and government personnel to move forward with whole-school-level changes (Bruun Jensen and Simovska, 2005).

### Youth engagement

In general, youth recognized the importance of embedding their perspectives in the school decision-making process. Most examples shared of youth engagement involved youth informing decisions rather than shared decision-making opportunities. Though the purpose of this study was not to specifically categorize responses based on Hart’s Ladder of Participation (Hart, 2008) on youth engagement, the findings seem to align with level five of the ladder—consultation and informing. This observation is similar to Larsson *et al.* (Larsson *et al.*, 2018) review related to children and young people’s participation in health and well-being interventions. Larsson *et al.* (Larsson *et al.*, 2018) highlighted that most interventions in schools involved students as informants rather than as collaborators or youth leaders. Specifically for youth engagement in whole-school health initiatives, Sprague Martinez *et al.* (Sprague Martinez *et al.*, 2020) outlined similar results indicating varying levels of youth engagement across schools in the USA with many of them also hovering around level five of Hart’s Ladder of Participation.

Despite the current study’s overall focus on HPS, the results in relation to youth engagement concerned their general involvement in school activities rather than specifically being engaged in activities related to school health promotion. This apparent disconnect may be due to the lack of engagement activities related to healthy school communities or the way in which the questions were written or asked by the peer researchers. Sprague Martinez *et al.* (Sprague Martinez *et al.*, 2018) shared that public health researchers have just recently began to embrace the importance of youth participation in advancing programme and intervention strategies, therefore this may partially explain the disconnect between youth engagement opportunities and school health promotion.

Findings related to youth engagement primarily focussed on what aided or impeded youth from being involved in school decision-making opportunities. Aligning with the Youth Engagement Framework (Busseri *et al.*, 2007), outlined in Rose-Krasnor (Rose-Krasnor, 2009), initiators of youth engagement were apparent at the individual, social and system level. Individually, youth passion helped to drive engagement, yet a supportive environment that encourages youth voice, and provides a range of opportunities to participate was suggested as a factor necessary to create meaningful youth engagement. Youth motivation to engage in decision-making processes was inhibited by a fear of speaking up due to beliefs that their opinions would be controversial, or not listened to or acted upon. Yet, if their voices were encouraged and heard they felt inspired to continue to be involved. This interpretation highlights a feedback cycle and the importance of a social environment that is supportive and safe, rather than judgemental and dismissive. The inter-relation of factors to promote or hinder youth engagement is consistent with past literature on initiators of youth engagement (Rose-Krasnor, 2009; Riemer *et al.*, 2014). The Youth Engagement Framework outlines similar initiators found in our research including values and interests (individual), adult and peer relationships (social) and community and cultural factors (system) (Rose-Krasnor, 2009). Particularly related to youth engagement in school health promotion, the Pan-Canadian Joint Consortium of School Health (Pan-Canadian Joint Consortium for School Health—Youth Engagement Toolkit, 2018) outlined eight qualities associated with youth engagement adapted from Eccles *et al.* (Eccles *et al.*, 2002) that aligned with our findings including factors such as safe spaces, supportive relationship, inclusion and nonjudgemental opportunities, being listened to and feeling like their voices mattered.

### Strengths and limitations

The major strength of this study was the use of YPAR to understand youth perspectives on HPS and school youth engagement. Further, by recruiting 23 youth from across different grade levels we obtained an array of perspectives on the topic of inquiry. Collecting qualitative research data from youth can readily be a challenge and require innovative approaches to sustain engagement. Involving youth as peer-researchers was a novel method to help to mitigate power dynamics between adult team members and youth, as well as provide more authentic participation from youth participants. However, peer researchers may have had limitations in interviewing techniques, such as probing or rephrasing of interview questions. Additionally, though the research team encouraged youth to interview a diverse sample of youth, no demographics besides grade and school were collected—therefore it is unknown the diversity of the population interviewed. The researcher who analysed the data (J.C.K.) has extensive experience in qualitative analysis, but was not involved in the interview process, and therefore lacked full familiarity with the data collected (Creswell and Poth, 2016). Lastly, the study did not focus on recruiting youth who attended schools that have formal youth engagement strategies embedded within their school communities. Future research will aim to replicate this project to specifically recruit youth who attend schools where youth engagement is embedded to examine if similarities and differences arise in the findings.

## CONCLUSION

This research provides unique insights into what youth think are important aspects of a healthy school community, and how they are engaged in school decision-making. Further, this study provides a useful example of how YPAR can be used to advance understanding of youth perspectives on factors related to school health promotion. With increasing recognition that youth engagement is a crucial part of HPS initiatives, this work provides relevant and applicable information on areas of importance for youth, as well as factors that may need to be promoted and mitigated to enhance a healthy school environment that places youth engagement at the forefront. These findings will have direct impact on progressing HPS initiatives in Nova Scotia, Canada by contributing to HPS youth engagement strategies, as well as offering transferrable information to advance school health initiatives elsewhere.

## Supplementary Material

daac081_suppl_Supplementary_Appendix

## Data Availability

The dataset supporting the results of this article is available from the authors upon reasonable request and the completion of a data transfer agreement.
