# Supplementary material for: Peering in: youth perspectives on Health Promoting Schools and youth engagement in Nova Scotia, Canada
Source: Health Promot Int. 2022 Jul 21;37(3):daac081. doi: 10.1093/heapro/daac081 (PMC9302890; doi:10.1093/heapro/daac081)
Supplement: daac081_suppl_Supplementary_Appendix [file daac081_suppl_supplementary_appendix.docx]

*Important things to remind your peers*

## The purpose of the interview/project

- - We are making recommendations to schools, government departments and other community members about how to improve youth engagement and leadership in creating healthy schools and communities in Nova Scotia. We can't make

meaningful recommendations without hearing from YOUTH

- - The research is also for Dalhousie University

## The risks/benefits and how you (the interviewer) will minimize those risks

**Risks**

- - The responses will be kept private, and we won’t tell their parents, teachers, or schools
  - The person being interviewed can stop the interview at any time

## Benefits

- - They will be part of influencing change from a youth perspective
  - The person being interviewed will get a gift card to thank them for their time

## Length of interview:

- - Give the person you are interviewing an idea about approximately how long the interview will take (1 hour)

## Safety:

- - Let the person you are interviewing know it is okay to say bad things about their school, the responses are anonymous, and we will never mention your peer’s name or their school’s name when reporting the results

Some notes for you:

The parts in this guide that are in *italics* are suggestions but it’s great if you say it in your own words so it’s comfortable for you.

Remember – the key areas we want to know about are:

1. How do these students describe a Healthy School Community?
2. What helps students have a voice, be part of decision making, and take meaningful action within schools and communities?
3. What are the issues related to health that are most important to these students?

The questions in this guide are meant to help find out their answers.

## Don’t forget!

You need to make sure to get their signed consent to participate form. This will have their address on it so we can send them a gift card for participating!

## Test Recording

As you begin each interview, it is a good idea to do a short test to make sure your voice recorder is working.

**CHECKLIST FOR EACH INTERVIEW**

- Collect completed consent from (check for name, signature, parent/guardian signature and address for gift card)
- Describe interview process to your peer and answer their questions about it
- START RECORDING
- Conduct interview
- STOP RECORDING
- Log interview and hours in your hours log

## KEY POINTS:

****START RECORDING****

# Introduction (about 2 min)

## Introduce yourself and thank them for being interviewed

- - - **Share why the interview is happening**

*Thank you for participating in this interview. My name is and I use the pronouns .*

*We are interested in learning about what young people think about how to make school communities healthier. We are especially interested in how your ideas and leadership can be a bigger part of making schools healthier, happier places - specifically in elementary and middle schools in the part of Mi’kmaq territory often called Nova Scotia.*

*What you share with us in this interview will make up some of the recommendations to government decision makers to make schools healthier places.*

*Your ideas are important, and so is your comfort participating. If at any point, you want to stop the interview, you can say so and we will.*

*I am going to record your answers on this voice recorder, so that the research team hears all of your ideas, and you may be quoted in the recommendations to government, but your name and identification will not be associated with the quote.*

*The whole interview should take about an hour.*

# Health Promoting Schools

*I am going you some questions about Health Promoting Schools. It’s okay if you don’t know what that means, we have this graphic to show you all the parts of Health Promoting Schools. I want to know what you think about all these different topics related to healthy schools. You don’t need to memorize them; you can look at this picture as we go along, or I’ll ask about the different topis as we go.*

What rules and guidelines support or get in the way?


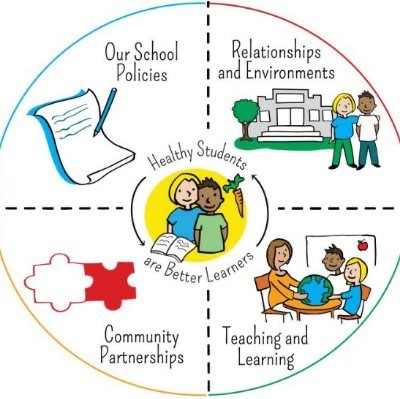


What are relationships like between students, and between students and staff?

What in the physical environment helps or makes it harder to be involved in making schools healthier places?

What organizations or people from outside the school help or make it harder for students to be part of change making?

What happens in classrooms that help students be part of changemaking and creating healthy schools?

## Healthy School Communities: what does it mean? (about 10 min)

*In this project we want to know what you think about the ways that your school can help you to be healthy and well.*

### Questions:

1. Now that you’ve seen this picture about healthy schools, tell me what a Healthy School Community means to you.
   - *Prompt different HPS topics, how do people act in a healthy school community?*
   - *How can you tell if a school community is healthy? (What does it look like? Feel like? What is happening)*
2. Do you think your school is a healthy community? Why/ why not?
   - *What is your school doing well to be a healthy school community?*
   - *What changes can make your school a healthier place?*
3. What people, organizations and business and other resources support your school to be a healthy environment?
   - *Give examples if you need- YMCA, etc.*

## What’s important to you? (approx. 10 min)

*In this section, we want to hear about what’s important to you related to health in schools*

### Questions

1. What grade and school are you going into this fall?
2. What are the biggest health and well-being issues and problems at your school?
   1. How are they being solved?
   2. And how would you solve it?
   3. How can young people help solve it or what can they do to solve it?
   4. What issues and challenges related to health and well-being in schools are most important to you?

## Youth Voice and Leadership (about 20 min)

*The research team, and adults in school health systems, say they want to find ways to make sure young people are part of making schools healthier places. This next section is to find out how* ***you*** *think this can happen*

### Questions:

1. Why is it important for schools to ask young people what they think about their school?
2. Have you had experiences (in your school) where you got to be part of decision making?
   1. How was this done?
   2. What did you like about the process? Why?
   3. Did you feel listened to?
   4. How would you improve it?
3. Do you know of any other ways students are part of decision making at your school?
   1. How was this done?
   2. What did you like about the process? Why?
   3. Did you feel listened to?
   4. How would you improve it?
4. How could students be more involved in decision making at your school?

- What inspires you and your peers to get involved?
- What gets in the way or makes it hard for you to be involved?
- What kind of support would make it easier to be more involved in decision making?

1. Describe a perfect world, where you feel like your ideas for a healthy school environment are respected and lead to positive change

*Use these prompts if you need:*

- *What is your role?*
- *What are adults doing?*
- *How often are you part of it?*
- *Who else should be there?*
- *What does the physical environment look like?*

## Closing (approx. 5 min)

1. Is there anything else you’d like to add?

*Thank you for sharing your ideas and your expertise! We are finishing all the interviews this summer and will be making recommendations to government this fall, based on what we find out from everyone involved. We can share a summary with you if you want to see what you contributed to and share it with your own community.*

*We also have a list of resources about health for young people on our website – if anything we talked about today makes you want to know or learn more, you can check it out at* [*www.upliftns.ca.*](http://www.upliftns.ca/) *There are also some links to help lines in case you or anyone you know needs that kind of support.*

*Thank you again – so much!*

*****STOP RECORDING*****
